# Preventing adverse drug reactions and more: current clinical use of pharmacogenetic testing

**DOI:** 10.1515/medgen-2025-2019

**Published:** 2025-07-17

**Authors:** Ursula Amstutz

**Affiliations:** Bern University Hospital Department of Clinical Chemistry and Clinical Genomics Lab, Inselspital Freiburgstrasse 3010 Bern Switzerland

**Keywords:** pharmacogenetics, pharmacogenomics, precision medicine, adverse drug reactions

## Abstract

Genomic variants affecting an individual’s response to drug therapies are common in populations worldwide. For clinical use, information on pharmacogenomic variation is translated into genotype-based therapy recommendations in evidence-based clinical practice guidelines for an increasing number of drugs. While clinical pharmacogenetic testing is currently still frequently performed at the level of individual genes, recent evidence highlighted the potential of a panel-based testing approach to substantially reduce the incidence of adverse drug reactions. This review provides background on pharmacogenetics, nomenclature used for reporting pharmacogenetic test results, as well as an overview of available resources for pharmacogenetic test interpretation.

## Introduction

An important goal of precision medicine is the identification of the optimal therapy based on the individual characteristics of a patient. In the context of drug therapies, this precision medicine approach aims to reduce inter-individual variability in drug response and safety. Both the lack of effectiveness as well as adverse drug reactions affect a significant proportion of patients for all major drug classes and provide an important burden to the health care system [1–4]. Besides somatic genomic variation being used to guide therapy selection for antineoplastic agents, pharmacogenetics is the field of precision medicine where inherited genetic variability is used for individual therapy optimization because it can affect the response to drug therapies. Such genetic variants are assessed using pharmacogenetic testing to identify patients with an increased risk of an inferior outcome to a given drug therapy [5], such as an increased risk of adverse drug effects or a reduced effectiveness. A recent study in the UK estimated that nine percent of adverse drug effects were related to drugs, for which the risk of the adverse effect could be modified by utilizing pharmacogenetic information to adjust the drug therapy [6].

## Mechanism of gene-drug interactions

**Figure 1:**
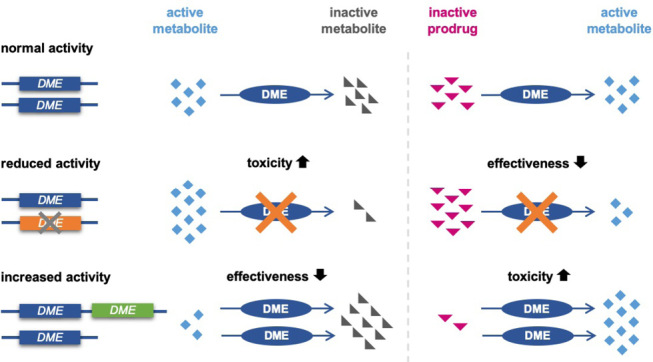
Differing effects of pharmacogenetic variants on the metabolism of different drugs. Left: effect of genetic variants on drugs inactivated by a drug metabolizing enzyme (DME). Right: effect of the same genetic variants on prodrugs converted into active metabolites by the same enzyme.

Pharmacogenomic variation can impact drug response through effects on pharmacokinetics, i.e. the absorption, distribution, metabolism and elimination of the drug, and by impacting interactions of the drug with its target or off-target effects (pharmacodynamics) [7, 8]. However, the majority of gene-drug interactions that are clinically well-established today are related to genes in drug metabolism pathways, thus impacting drug pharmacokinetics. In addition, immune-mediated adverse drug reactions are associated with specific *HLA* alleles [5]. Given that drug metabolizing enzymes such as those of the Cytochrome P450 family can have relatively broad substrate specificities, some pharmacogenes affect the pharmacokinetics of multiple drugs or drug classes. As a consequence, the same pharmacogenetic variant can have different effects the pharmacokinetics of a pharmacologically active compound, depending on the role of the encoded enzyme in the metabolism of the respective drug. In more detail, a genetically based reduction in the activity of an enzyme performing the bioactivation of a prodrug into its pharmacologically active form will result in a reduced pharmacological effect of that drug (Figure 1). For another drug, the same enzyme may convert the pharmacologically active compound into an inactive metabolite. The same pharmacogenetic variant causing reduced enzyme activity can thus result in an increased risk of adverse drug reactions for this drug due to a decreased catabolism of the pharmacologically active compound [7]. Examples of such drug metabolizing enzymes are Cytochrome P450 2D6 (CYP2D6), converting prodrugs such as codeine or tamoxifen into pharmacologically more active compounds as well as catabolizing tricyclic antidepressants and selective serotonin reuptake inhibitors into inactive metabolites, or CYP2C19 converting the antiplatelet drug clopidogrel into its active metabolite while inactivating other drugs such as antidepressants or proton pump inhibitors [9, 10].

## Abundance of pharmacogenetic variation

Contrary to pathogenic germline variants causing monogenetic diseases, pharmacogenetic variants are not associated with a disease phenotype, and therefore can be common in the population. The high frequencies of these variants are likely due to their lack of a strong negative impact on health in the absence of certain pharmacological agents, and thus also a lack of negative selection. For example, variation in the *CYP2D6* gene encoding the drug metabolizing enzyme CYP2D6 is so common that a majority of individuals in some global populations, including Europeans, carry at least one allele associated with altered enzyme activity [11]. The spectrum of genetic variation in pharmacogenes is diverse and includes copy number variation (full gene deletions and duplications, deletions of individual exons), splice variants, regulatory variants as well as coding single nucleotide variants (SNVs) with varying impact on enzyme activity. Again, *CYP2D6* is a prime example with variation in this gene encompassing whole gene deletions, duplication, multiplication, splice variants, haplotypes containing multiple nonsynonymous coding SNVs, as well as hybrid alleles combining sequences from *CYP2D6* and the pseudogene *CYP2D7* [12].

Due to the abundance of pharmacogenetic variants and the substantial number of drugs affected, including many frequently used medications, pharmacogenetic effects are not a rare occurrence. Even if for a specific drug only a minority of individuals are expected to be “therapeutic outliers” at risk of suboptimal outcomes due to a pharmacogenetic variant, the cumulative impact across all currently known gene-drug interactions is much higher. In fact, it has been shown in various populations, including Caucasians, that over 90 % of individuals carry in at least one pharmacogene one or more clinically actionable genotypes or diplotypes, i.e. a genotype or diplotype for which a therapy change such as dose adjustment or alternative drug is recommended [5]. Similarly, exposure to drugs with known pharmacogenomic effects is high. A study in the UK estimated that 89 % of patients aged ≥70 year were prescribed at least one drug affected by pharmacogenomic variability over the previous 20 years [13]. Even among individuals aged 50–59 years, this number was high at 71 %.

## Genotype nomenclature

Due to the presence of numerous common coding SNVs within pharmacogenes such as *CYP2D6*, a nomenclature designating full-gene haplotypes using star (*) alleles is used to describe pharmacogenetic test results in those genes with sufficiently strong linkage disequilibrium across the relevant genomic region [14]. For *CYP2D6*, over 170 different star (*) alleles have been recorded so far. With this nomenclature, the allele *1 generally designates the reference allele. Of note, in many pharmacogenetic assays used in clinical laboratories, only a selected number of SNVs are genotyped for a given gene. In this context, assignment of the *1 allele is used to designate absence of non-reference alleles in the genotyped SNVs. For genotyping methods that do not include resequencing of all relevant genomic regions, a *1 allele assignment, while excluding other common alleles, may thus not represent a true reference allele as information on ungenotyped rare variants is not available. For several pharmacogenes, recommendations on which alleles should be included in genotyping assays are available from the Association of Molecular Pathology PGx Working group in conjunction with other expert groups, grouped as “tier 1” for a minimum set of variants and “tier 2” for an extended panel [15].

A notable exception to the (*) allele nomenclature is the dihydropyrimidine dehydrogenase gene (*DPYD*) relevant for identifying patients at risk of severe fluoropyrimidine-related toxicity. Due to the size of this gene encompassing 950 kb and multiple regions of low linkage disequilibrium, the inference of full-gene haplotypes based on genotypes of individual SNVs, as is commonly done for other pharmacogenes, is not possible [16]. For this gene, pharmacogenetic results are thus reported using genotypes for individual SNVs.

## Phenotype nomenclature

Given the high population frequency of many pharmacogenomic variants, functional characterization of numerous alleles has been performed by measurements of in vivo drug metabolic ratios or in vitro enzyme activity. Based on this functional characterization, alleles are designated as normal, increased, decreased or no function [17]. “Normal function” describes a fully functional “wild-type” allele, while increased function is used for alleles that have greater than normal function or activity. Increased function alleles include duplications or multiplications of fully functional alleles, but also alleles carrying regulatory variants resulting in increased gene expression. “Reduced function” is used to describe alleles with reduced function compared to normal function alleles, but that still have some residual function or activity, whereas “no function” is used for alleles that are fully nonfunctional or have only minimal residual activity. Such “no function” alleles may include full gene deletions, alleles with splice variants, but also coding SNVs with a strongly detrimental impact on enzyme activity.

Based on the function of individual alleles, a genotype-based predicted phenotype is assigned based on the allele diplotype. For phenotype assignment, an additive approach is commonly used to combine the effect of both alleles, grouping phenotypes as “normal” (NM), “intermediate” (IM), “poor” (PM), “rapid” (RM), and “ultrarapid” (UM) metabolizers for drug metabolizing enzymes [17]. With this classification, PM generally designates little to no predicted enzyme activity, and IM a decreased activity intermediate between NM and PM. The UM classification is used for increased activity compared to NM. RM is used for increased activity compared to NM, but lower activity compared to UM. For example, RM is used for heterozygous genotypes of an increased expression allele of *CYP2C19* (*1/*17), whereas UM is used for homozygous carriers of the same allele (*17/*17).

The phenotype assignment based on diplotypes of functionally characterized alleles is defined for each gene by expert groups such as the Clinical Pharmacogenetics Implementation Consortium (CPIC) [18]. To this aim, a numeric assignment of allele function is used with a value of 1 for “normal function” alleles, 0.25–0.5 for “reduced function” alleles, 0 for “no function” alleles, and >1 for “increased function” alleles. The addition of the scores for each individual allele yields the overall activity score of a diplotype. The cutoffs, based on which the diplotype activity score is grouped e.g. as “normal” or “intermediate” metabolizer can vary between different genes. For example, for *CYP2D6*, an activity score of 1.5 is classified as NM, whereas the same activity score is classified as IM for *DPYD*. Of note, genotype-based therapy recommendations may differ not only between phenotype groups, but also within a metabolizer group. For example, for *CYP2C9*, activity scores of 1.5 and 1 are both designated as IM, but therapy adjustments such as dose reductions are recommended only for diplotypes with an activity score of 1, but not diplotypes with an activity score of 1.5, for some drugs [19].

## Decision support for clinical use of pharmacogenetics

**Figure 2.**
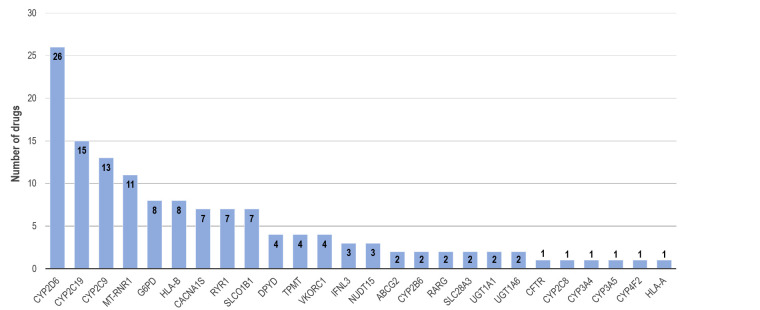
Number of drugs per gene in pharmacogenetic clinical practice guidelines listed on PharmGKB. Drugs included in guidelines but without genotype-specific therapy recommendation were excluded.

To facilitate the use of pharmacogenomic information in clinical practice, guidelines have been developed by international and national expert groups such as CPIC, the Dutch Pharmacogenetic Working Group (DPWG) or the French National Network of Pharmacogenetics (RNPGx). These guidelines are based on evidence from the literature of clinical studies and expert consensus and provide recommendations on how a drug therapy should be adjusted based on a pharmacogenetic test result, including genotype-specific dosing recommendations or use of alternative therapies. Many of these guidelines are compiled and summarized in the Pharmacogenomics Knowledgebase PharmGKB (www.pharmgkb.org) [10, 20]. At the time of writing, CPIC guidelines with genotype-based therapy recommendations were available for 79 drugs, DPWG recommendations for 53 drugs, with 24 additional guidelines from other groups. Overall, pharmacogenetic guidelines were available from at least one expert group for a total of 108 drugs. Of note, the number of genes included in these guidelines is considerably smaller with a total of 26 genes (Table 1). This reflects the broad substrate specificity of some drug metabolizing enzymes, making the same gene relevant for a number of different drugs. In particular, the number of drugs is highest for the *CYP2D6*, *CYP2C19* and *CYP2C9* genes (Figure 2).

PharmGKB also compiles pharmacogenetic information from drug labels, making it a very comprehensive resource for clinical use of pharmacogenetic testing. To date, PharmGKB includes drug label annotations by the US Food and Drug Administration (FDA), European Medicines Agency (EMA) or Health Canada (HCSC) on germline pharmacogenetics for 169 drugs. Of these annotations, a recommendation or requirement for pharmacogenetic testing is included in the label of at least one of these three agencies for 24 drugs and ten different genes (Table 2). While regulatory agencies are in agreement for some gene-drug combinations, there are also discrepancies such as for the cardiac myosin inhibitor mavacamten, where FDA and HCSC only include information about pharmacogenetic effects whereas the EMA label includes a recommendation for testing.

**Table 1.** Gene-drug combinations with recommendations for genotype-guided therapy adjustment provided in clinical practice guidelines listed on PharmGKB. Genes included in the pharmacogenetic panel of the PREPARE trial [21] are indicated in bold.

Gene	Drugs
*ABCG2*	allopurinol, rosuvastatin
*CACNA1S*	desflurane, enflurane, halothane, isoflurane, methoxyflurane, sevoflurane, succinylcholine
*CFTR*	ivacaftor
***CYP2B6***	efavirenz, sertraline
***CYP2C19***	amitriptyline, citalopram, clomipramine, clopidogrel, dexlansoprazole, doxepin, escitalopram, imipramine, lansoprazole, mavacamten, omeprazole, pantoprazole, sertraline, trimipramine, voriconazole
*CYP2C8*	ibuprofen
***CYP2C9***	acenocoumarol, celecoxib, fluindione, flurbiprofen, fosphenytoin, ibuprofen, lornoxicam, meloxicam, phenytoin, piroxicam, siponimod, tenoxicam, warfarin
***CYP2D6***	amitriptyline, aripiprazole, atomoxetine, brexpiprazole, clomipramine, codeine, desipramine, doxepin, eliglustat, flecainide, haloperidol, hydrocodone, imipramine, metoprolol, nortriptyline, ondansetron, paroxetine, pimozide, propafenone, risperidone, tamoxifen, tramadol, trimipramine, tropisetron, venlafaxine, zuclopenthixol
*CYP3A4*	quetiapine
***CYP3A5***	tacrolimus
*CYP4F2*	warfarin
***DPYD***	capecitabine, flucytosine, fluorouracil, tegafur
*G6PD*	dapsone, methylene blue, nitrofurantoin, pegloticase, primaquine, rasburicase, tafenoquine, toluidine blue
*HLA-A*	carbamazepine
***HLA-B***	abacavir, allopurinol, carbamazepine, flucloxacillin, fosphenytoin, lamotrigine, oxcarbazepine, phenytoin
*IFNL3*	peginterferon alfa–2a, peginterferon alfa–2b, ribavirin
*MT-RNR1*	amikacin, dibekacin, gentamicin, kanamycin, neomycin, netilmicin, paromomycin, plazomicin, ribostamycin, streptomycin, tobramycin
*NUDT15*	azathioprine, mercaptopurine, thioguanine
*RARG*	daunorubicin, doxorubicin
*RYR1*	desflurane, enflurane, halothane, isoflurane, methoxyflurane, sevoflurane, succinylcholine
*SLC28A3*	daunorubicin, doxorubicin
***SLCO1B1***	atorvastatin, hmg coa reductase inhibitors, lovastatin, pitavastatin, pravastatin, rosuvastatin, simvastatin
***TPMT***	azathioprine, cisplatin, mercaptopurine, thioguanine
***UGT1A1***	atazanavir, irinotecan
*UGT1A6*	daunorubicin, doxorubicin
***VKORC1***	acenocoumarol, fluindione, phenprocoumon, warfarin

**Table 2.** Gene-drug combinations with a recommendation or requirement for pharmacogenetic testing in a health regulatory agency label (FDA: US Food and Drug Administration; EMA: European Medicines Agency; HCSC: Health Canada).

Gene	Drug	Indication	FDA	EMA	HCSC
APOE	aducanumab	mild cognitive impairment, mild dementia	req	n/a	n/a
	lecanemab	mild cognitive impairment, mild dementia	req	n/a	n/a
CYP2C19	mavacamten	heart failure	info	req	info
CYP2C9	siponimod	multiple sclerosis	req	req	req
CYP2D6	dextromethorphan/ quinidine	Pseudobulbar affect in amyotrophic lateral sclerosis or multiple sclerosis	rec	no	n/a
	eliglustat	Gaucher’s disease	req	req	req
	pimozide	Tourette’s disorder	req	n/a	n/a
	tetrabenazine	Chorea Huntington	req	n/a	act
DPYD	capecitabine	various solid tumours	rec	rec	rec
	fluorouracil	various solid tumours	rec	rec^a^	rec
	tegafur / gimeracil / oteracil	gastric, colorectal cancer	n/a	rec	n/a
G6PD	pegloticase	gout	req	req	n/a
	primaquine	malaria	req	n/a	req
	tafenoquine	malaria	req	n/a	n/a
	rasburicase	chemotherapy-induced hyperuricemia	req	act	req
HLA-A	tebentafusp	uveal melanoma	req	req	req
HLA-A, HLA-B	carbamazepine	epilepsy, trigeminal neuralgia	act, req	n/a	rec
	oxcarbazepine	epilepsy	rec	n/a	rec
HLA-B	abacavir	HIV	req	req	req
	allopurinol	gout	rec	n/a	rec
TPMT	azathioprine	immunosuppression	rec	n/a	req
	mercaptopurine	acute lymphocytic/myeloid leukemia	rec	rec	rec
	thioguanine	acute lymphocytic/myeloid leukemia	rec	n/a	rec
UGT1A1	irinotecan	various solid tumours	rec	act	rec
Abbreviations: req = testing required; rec = testing recommended; act = actionable information (dosing information, contraindication, alternate drug or other guidance for specific genotype or phenotype); info = information on effect of a genotype/phenotype without further guidance; n/a = no label information found ^a^separate recommendation issued by EMA, not referenced in PharmGKB					

## Clinical implementation

The impact of a regulatory agency issuing a recommendation for pharmacogenetic testing was demonstrated for *DPYD* testing in patients prior to receiving fluoropyrimidine-based chemotherapy. In 2020, the EMA issued a recommendation to test for reduced dihydropyrimidine dehydrogenase (DPD) enzyme activity either by genotype- or phenotype prior to administering chemotherapy including fluorouracil, capecitabine or tegafur [22]. DPD is the first and rate-limiting enzyme in the catabolism of 5-fluorouracil. 5-fluorouracil and its orally administered prodrug capecitabine are commonly used in chemotherapy of various solid tumours including colorectal and other gastric cancers, as well as breast cancer. Several variants in the dihydropyrimidine dehydrogenase gene *DPYD* are associated with reduced DPD activity and an increased risk of severe to life-threatening toxicity from standard doses of fluoropyrimidine-based chemotherapy [23]. Using pre-emptive genotyping to identify patients carrying *DPYD* risk variants and administering a reduced dose in these patients has been shown to reduce the risk of severe toxicity [24, 25]. While evidence for the clinical benefit and cost-effectiveness of *DPYD* testing was available in the scientific literature several years earlier, widespread uptake throughout Europe primarily took place after the EMA recommendation and similar recommendations by oncology societies [26] were published [27, 28].

The pharmacogenetic test with the earliest widespread clinical implementation was HLA-B*57:01 testing to prevent abacavir-induced hypersensitivity reactions. Prior to the implementation of this test, hypersensitivity reactions including fever, rash, gastrointestinal and respiratory symptoms were observed in 5 to 8 % of patients during the first weeks of therapy. A strong association of HLA-B*57:01 with an increased risk of abacavir hypersensitivity was identified in association studies [29, 30], and a subsequent randomized trial demonstrated a strong reduction in hypersensitivity reactions with pre-emptive HLA-B*57:01 testing [31]. Following these findings, associations of HLA alleles with multiple other immune-mediated adverse drug reactions were identified, including cutaneous adverse drug reactions (e.g. HLA-B*15:02, HLA-A*31:01 for carbamazepine and phenytoin, HLA-B*58:01 for allopurinol) and drug-induced liver injury (e.g. HLA-B*57:01 for flucloxacillin) [5]. While these findings provided important insights into the pathomechanisms underlying theses adverse drug reactions [32], testing for these *HLA* variants has not found as widespread clinical uptake as for HLA-B*57:01 in the context of abacavir therapy.

On one hand, this can be attributed to regional differences in the population frequency of these alleles. While the overall prevalence of actionable pharmacogenomic variation is ubiquitously high, there is substantial variability in the population frequencies of individual pharmacogenetic variants [11], which can result in differing performance of an individual pharmacogenetic test depending on a patient’s genetic ancestry and the alleles included in a test. In drug metabolism pharmacogenes such as *DPYD*, *TPMT* and *UGT1A1*, different clinically relevant variants have higher frequencies in African or Asian populations compared to Europeans. As a consequence, a pharmacogenetic test that only includes risk variants common in Europeans will identify a lower proportion of patients at increased risk of drug toxicity in some patients with non-European ancestry. Additionally, for pharmacogenetic variants with variable population frequencies such as individual *HLA* alleles, the cost-effectiveness of an individual test can vary due to the impact of the variant frequency on the pre- and post-test probabilities resulting in a higher number needed to test (NNT) to identify an individual with an actionable test result in patients from populations where the frequency of the risk allele is low [5].

This limitation in cost-effectiveness of testing for low frequency variants with high NNT can be mitigated by using a panel-based approach combining the analysis of the most relevant pharmacogenes in a single test. Such an approach was recently applied in the large cluster-randomized crossover implementation trial (PREPARE) conducted in multiple European countries with 6944 participants [21]. In this trial, a 12-gene pharmacogenetic panel test (Table 1) including 50 variants was used in all patients with a prescription for a drug with a pharmacogenetic guideline recommendation for a gene in the panel. In the intervention group, the test result was communicated to the treating physicians – who additionally underwent a pharmacogenetic training how to transform pharmacogenetic diagnostics into clinical decisions – without a mandate to follow the genotype-guided therapy recommendation. Overall, a 25 % reduction in the incidence of adverse drug reactions was observed in the intervention group (21.5 %) compared to the control group receiving standard treatment (28.6 %) with an odds ratio of 0.7 (95 % CI: 0.61–0.79) [21]. This study thus clearly demonstrated both the clinical benefit of pharmacogenotype-guided adjustment of drug therapies performed by adequately trained physicians, as well as the potential of a pre-emptive panel-based pharmacogenetic testing approach.

Of note, the reduction in the incidence of adverse drug reactions was similar in an analysis including all patients, and in an analysis only including patients with actionable genotype. This was unexpected as an impact of pharmacogenetic testing was primarily expected in patients, in whom the test result was associated with a recommendation for a change in the drug therapy. However, this result can be explained by additional statistical analyses that considered the patient mix. In more detail, due to the real-world design and the study taking place during the COVID pandemic, additional study sites had to be added during the course of the study to ensure sufficient recruitment. As a consequence, given the cross-over design with randomization by study country, and each country switching either from control to intervention, or from intervention to control after half of the study duration, the mix of patients differed between the intervention and the control group. Accounting for this difference in the case mix, and the associated differences in the drugs the patients received, in the statistical analysis resulted in an increased effect of the pharmacogenetic intervention in the patients with actionable genotype (odds ratio = 0.61, 95 % CI: 0.51–0.74), and a reduced effect in patients without actionable genotype (odds ratio = 0.87, 95 % CI: 0.67–1.12).

## Multifactorial nature of variable drug response

In addition to variable population frequencies, the positive predictive value of testing for *HLA* alleles associated with immune-mediated adverse drug reactions can also be very low due to their relatively common occurrence in the population, while the incidence of the associated adverse drug reaction is much lower [33]. This relatively low positive predictive value of some pharmacogenetic associations, i.e. only a proportion of carriers of a risk variant developing an adverse outcome when being administered the drug in question, highlights the multifactorial nature of variability in drug response. While pharmacogenomic variation is an important contributor to this variability for some drug therapies, for most of these drugs, the pharmacogenetic effects only account for a part of the overall variability. In the example of *HLA*-associated adverse drug reactions, while the presence of certain *HLA* alleles can essentially be considered a requirement for the adverse event to occur, their presence alone is not sufficient. In addition to a given *HLA* allele, other factors thus likely need to be present in a patient in order to trigger the drug-induced immune reaction. Similarly, for drug metabolism-related pharmacogenetic effects such as those of CYP genes or *DPYD*, within patients with the same genotype, there is substantial remaining variability in the observed enzyme activity unexplained by the currently known genetic variation [25, 34]. This results, for example, in some carriers of *DPYD* risk variants tolerating normal doses of fluoropyrimidines.

Importantly, this limitation does not eliminate the clinical benefit of pharmacogenetics-informed drug therapy adjustment as illustrated in the example of *DPYD* testing [24, 25] or the results of the PREPARE trial [21]. Awareness of remaining variability unexplained by current pharmacogenetic variants is, however, important to optimally use pharmacogenomic information in clinical practice. For genetic effects related to drug metabolism, therapeutic drug monitoring provides a useful complement to pharmacogenetic testing for drugs with a defined therapeutic window. For infusional 5-fluorouracil, an area under the curve (AUC) of 20–30 mg*h/L has been identified as an effective drug concentration while minimizing adverse effects, and is used as a target for therapeutic drug monitoring-based dose adjustment [35, 36]. To maximize therapy effectiveness in carriers of *DPYD* risk variants, in whom a 50 % reduction of the starting dose is recommended, therapeutic drug monitoring can be used to identify patients with subtherapeutic drug exposure following this initial dose reduction, allowing for dose titration in subsequent chemotherapy cycles [37]. As therapeutic drug monitoring has not been established for the oral prodrug of 5-fluorouracil, capecitabine, use of infusional 5-fluorouracil instead of capecitabine may thus be preferable in *DPYD* risk variant carriers [37]. Similarly, therapeutic drug monitoring is available for several medications metabolized by CYP enzymes such as CYP2D6 or CYP2C19 [38, 39].

Additionally, drug-drug interactions may add non-genetically determined variability in the activity of these enzymes and need to be considered. Such drug-drug interactions can lead to a mismatch between the predicted drug metabolism phenotype based on an individual’s genotype and the observed drug metabolism (phenoconversion) [40]. For example, an individual heterozygous for a non-functional *CYP2D6* allele (intermediate metabolizer based on genotype) concomitantly receiving a CYP2D6 inhibitor may have a CYP2D6 activity similar to individuals with two nonfunctional *CYP2D6* alleles, i.e. a poor metabolizer phenotype [41]. In addition to drug-drug interactions, phenoconversion may also occur as a result of comorbidities such as inflammation, smoking or pregnancy [41]. One example, where phenoconversion may have obscured pharmacogenetic effects is the antiestrogen tamoxifen used for the treatment of breast cancer. CYP2D6 is involved in the conversion of tamoxifen to endoxifen, its most abundant pharmacologically active metabolite. Breast cancer patients with *CYP2D6* poor metabolizer genotypes have been shown to have lower endoxifen concentrations and a reduced disease-free survival in some studies, while others did not observe such an effect [42]. While other factors such as genotyping comprehensiveness (i.e. genotype test not including relevant PM alleles) or the use of tumour tissue with loss-of-heterozygosity at the *CYP2D6* locus [43] also contributed to discrepancies between study findings as demonstrated by a recent meta-analysis [44], the lack of incorporation of CYP2D6 inhibitor usage may have added further heterogeneity [42].

Given this interplay between drug-gene and drug-drug interactions, the CPIC guidelines for *CYP2D6* genotype and tamoxifen therapy also include recommendations regarding use of CYP2D6 inhibitors in different genotype groups. Unfortunately, however, most clinical practice guidelines on pharmacogenetic testing or drug-drug interactions available today only provide recommendations related to either gene-drug or drug-drug effects. However, phenotypic models of drug-drug-gene-interactions have recently become available, which could provide guidance for the combination of both [45]. There is thus potential to further improve dose or therapy optimization by combining information on multiple factors influencing drug pharmacokinetics or -dynamics. In the future, drug dose or therapy selection should ideally be guided by consideration of relevant genotypes and concomitant medications, but also sex differences which currently result in diversity in the risk of adverse drug reactions between men and women, and other patient information affecting vulnerability to adverse drug reactions [46].

## Conclusions and outlook

While testing of individual pharmacogenes has already become part of routine clinical practice for some gene-drug pairs such das *DPYD* and fluoropyrimidines, HLA-B*57:01 and abacavir, as well as *TPMT* and thiopurines, recent evidence such as the PREPARE trial highlights the potential of a broader pharmacogenetic testing approach for pharmacogenetic variants that are “actionable” because drug therapy can be adjusted to reduce adverse drug reactions or negative therapeutic outcome. Similar to genomic testing for monogenetic diseases, where single-gene testing has largely been replaced by panel or whole exome sequencing, simultaneous testing of multiple pharmacogenes in a panel-based approach will likely reduce or eliminate some of the barriers to a more wide-spread use of pharmcogenetic testing related to numbers needed to test and limited cost-effectiveness of single-gene tests. Additionally, use of pre-emptive pharmacogenetic panel testing will make genomic results of relevance for drug therapies readily available for medications that need to be administered in emergency settings such as antiplatelet therapy for acute coronary syndrome [47]. Key to optimal utilization of panel-based pharmcogenomic testing is thus not only the testing approach, but also the availability of pharmacogenetic results for therapy decisions. Specifically, long-term availability of pharmacogenetic testing results would be required beyond the original indication in electronic health care records or as a kind of pharmacogenetic passport [48] to ensure that pharmaco-genotypes are available to treating physicians when prescribing other drugs later on in a patient’s life.

Furthermore, while the frequency of known pharmacogenetic variants has been assessed in populations worldwide [11], much of the knowledge on gene-drug interactions available today is based on patients of European ancestry. Studies of pharmacogenomic variation, its potential functional impact and association with drug therapies in patients of other ancestries, similar to a recent large-scale study in Chinese individuals [49], will be key to ensure that genotyping panels include relevant variants so that all patients can equally benefit from pharmacogenetics-based drug therapy optimization.

Finally, while current testing methodologies commonly utilize real-time PCR based genotyping of individual SNVs or short-read sequencing, long-read high-throughput sequencing offers substantial potential to improve pharmacogenetic testing at the analytical level. By allowing for the detection of rare variants and the direct analysis of gametic phase [50, 51], long-read sequencing can improve the resolution of pharmacogenetic results, particularly for patients with ancestry in understudied populations where different variants may be prevalent and haplotype structures may differ. With the increased utilization of long-read whole genome sequencing in medical genetics, as well as National sequencing programs such as the model project genome sequencing in Germany, a pharmacogenomic interrogation of these sequencing data and incorporation of pharmacogene diplotype information into patients’ medical records should thus be integrated into the testing workflow. To this aim, inter-disciplinary collaboration between medical geneticists and clinical pharmacologists (the only speciality in medicine where pharmacogenetics is part of the speciality training) as pharmacogenomics experts will be of importance in order to utilize available genomic information for the optimal benefit of patients.
